# High-throughput screening identifies a novel natural product-inspired scaffold capable of inhibiting *Clostridioides difficile* in vitro

**DOI:** 10.1038/s41598-021-90314-3

**Published:** 2021-05-25

**Authors:** Rusha Pal, Mingji Dai, Mohamed N. Seleem

**Affiliations:** 1 Department of Comparative Pathobiology, College of Veterinary Medicine, Purdue University, West Lafayette, IN 47907 USA; 2 Department of Biomedical Sciences and Pathobiology, Virginia-Maryland College of Veterinary Medicine, Virginia Polytechnic Institute and State University, Blacksburg, VA 24061 USA; 3 Department of Chemistry and Center for Cancer Research, Purdue University, West Lafayette, IN 47907 USA; 4 Center for Emerging, Zoonotic and Arthropod-Borne Pathogens, Virginia Polytechnic Institute and State University, Blacksburg, VA 24061 USA

**Keywords:** Drug discovery, Microbiology

## Abstract

*Clostridioides difficile* is an enteric pathogen responsible for causing debilitating diarrhea, mostly in hospitalized patients. The bacterium exploits on microbial dysbiosis induced by the use of antibiotics to establish infection that ranges from mild watery diarrhea to pseudomembranous colitis. The increased prevalence of the disease accompanied by exacerbated comorbidity and the paucity of anticlostridial drugs that can tackle recurrence entails novel therapeutic options. Here, we report new lead molecules with potent anticlostridial activity from the AnalytiCon NATx library featuring natural product-inspired or natural product-derived small molecules. A high-throughput whole-cell-based screening of 5000 synthetic compounds from the AnalytiCon NATx library helped us identify 10 compounds capable of inhibiting the pathogen. Out of these 10 hits, we found 3 compounds with potent activity against *C. difficile* (MIC = 0.5–2 μg/ml). Interestingly, these compounds had minimal to no effect on the indigenous intestinal microbial species tested, unlike the standard-of-care antibiotics vancomycin and fidaxomicin. Further in vitro investigation revealed that the compounds were nontoxic to Caco-2 cell line. Given their potent anticlostridial activity, natural product-inspired scaffolds may suggest potential avenues that can address the unmet needs in preventing *C. difficile* mediated disease.

## Introduction

*Clostridioides* (*Clostridium*) *difficile* is a leading cause of nosocomial diarrhea worldwide^1,2^. The bacterium has been identified and classified as an urgent threat by the Centers for Disease Control and Prevention (CDC) with an estimated 223,900 cases of *C. difficile* infection (CDI) in hospitalized patients and 12,800 deaths in the year of 2019^3^. The clinical manifestations of CDI range from mild diarrhea to fulminant infection which can involve toxic megacolon, bowel perforation, sepsis, and even death^1,2,4^.

Treatment with antibiotics constitutes the foremost risk factor of CDI. Antibiotic use leads to a perturbation in the diversity of the host microflora and its related metabolome. The disruption of the host microbiome enables *C. difficile* outgrowth and colonization in the intestine which is ensued by the secretion of toxins^2^. These gut-damaging clostridial toxins mainly include two large homologous toxins, toxin A (TcdA) and toxin B (TcdB), which are the primary determinants of disease pathogenesis that manifest as watery diarrhea or develop into fatal gastrointestinal sequelae like pseudomembranous and fulminant colitis^5^.

Albeit the fact that antibiotics typically incite CDI, the clinical armamentarium for CDI is limited to antibiotics vancomycin, metronidazole, and fidaxomicin^6^. Metronidazole was initially used for the treatment of non-severe CDI. However, Infectious Disease Society of America (ISDA) and the Society for Healthcare Epidemiology of America (SHEA) now recommends the use of vancomycin and fidaxomicin over metronidazole for the treatment of initial episode^6^. The rate of clinical cure associated with the use of these antibiotics range between 72 and 81% with patients diagnosed for the first time having an approximate 20% likelihood of recurrence^7,8^. Following the first recurrence event, the risk of subsequent recurrences can increase by up to 50%^9^. The unprecedented challenges associated with the current treatment regime calls for an avant-garde drug scaffold that has the potential to treat CDI.

An important component of modern drug discovery, high-throughput screening (HTS) is a keystone technology used to identify novel chemical entities that has the potential to become usable drugs^10,11^. De novo drug discovery, which focuses on the identification of innovative chemical scaffolds, integrates discovery based on either target-based HTS screening (screening to identify inhibitors of a specific enzyme target) or whole-cell-based phenotypic HTS screening (screening against a whole organism)^11^. In this study, a whole-cell-based high throughput screening of the AnalytiCon NATx library (consisting of 5000 natural product-inspired or natural product-derived synthetic compounds) was conducted with the goal of identifying novel scaffolds that have the potential to treat CDI. Unlike most of the complex natural products, the natural product-like synthetic compounds in the NATx compound library are prepared by reliable chemistry and are suitable for further medicinal chemistry optimization. Here, among the panel of hits identified that could inhibit the growth of *C. difficile*, we identified molecules with potent anti *C. difficile* activity. Minimum inhibitory concentration (MIC) of the hit compounds was determined against representative members of the human gut microflora. We also investigated the hit scaffolds for their cytotoxicity against human colorectal adenocarcinoma cell line (Caco-2).

## Results

### High-throughput screen of AnalytiCon NATx library and validation of hits using plate cherry-picking against *C. difficile* ATCC BAA 1870

**Figure 1:**
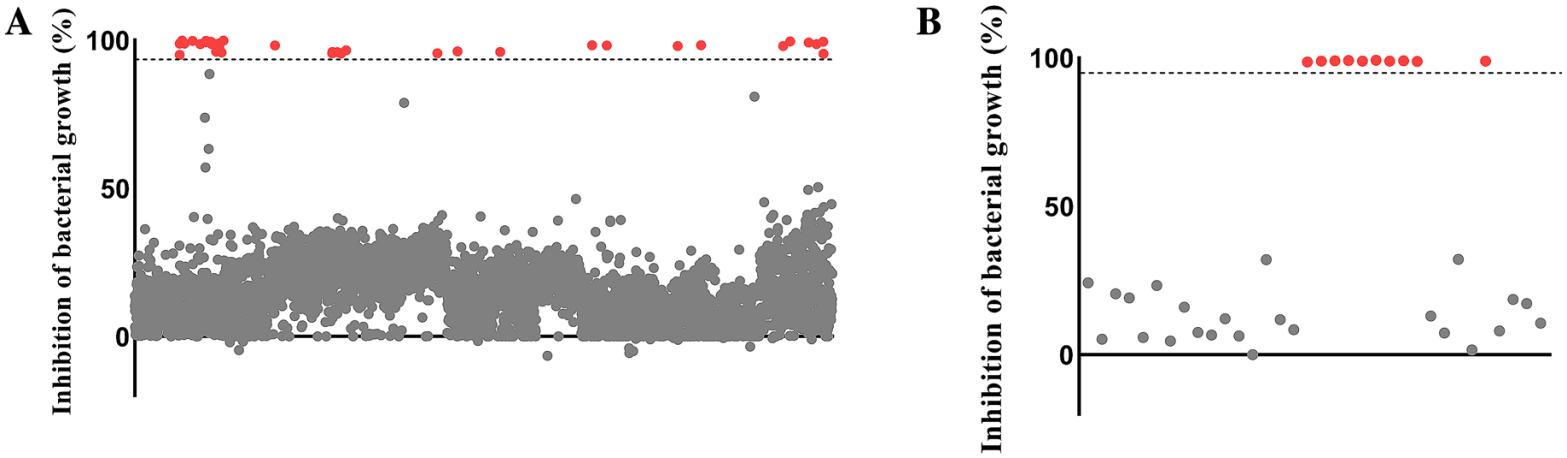
High-through screening (HTS) of the AnalytiCon NATx library identifies novel lead scaffolds. (**A**) Results from HTS of 5000 natural product-like compounds from the AnalytiCon NATx library. Compounds were screened at a concentration of 3 µM against *C. difficile* ATCC BAA 1870. Compounds exhibiting greater than 95% inhibition of bacterial growth were deemed to be hits. 34 hits were obtained from the initial screening of the library. (**B**) Plate cherry-picking of the 34 hits obtained from the initial screening revealed a final count of 10 hits against *C. difficile* ATCC BAA 1870 at a concentration of 3 µM.

The AnalytiCon NATx library containing 5000 natural product-like synthetic compounds was screened for possible inhibitors of *C. difficile* at a concentration of 3 $\upmu$M. In the initial screening, we obtained 34 compounds out of the 5000 that inhibited the growth of the pathogen (Fig. 1A). In order to confirm the anticlostridial activity of the hit compounds, the 34 hit compounds were cherry-picked from the plates and rescreened against *C. difficile* at the same concentration. The plate cherry-picking confirmed the anticlostridial activity of 10 compounds from the initially obtained 34 hits (Fig. 1B)*.*

### Antibacterial activity of the hit scaffolds against a panel of *C. difficile* strains

MIC assays were performed to determine the spectrum of inhibitory effects of the hit scaffolds against a panel of *C. difficile* strains (Table S2). As shown in Table 1A, 3 compounds (NAT13-338148, NAT18-355531, and NAT18-355768) could inhibit the bacteria at concentrations ranging from 0.5–2 $\upmu$g/ml. NAT5-397881, NAT13-331545, NAT18-356312, NAT27-401005, NAT27-401503, and NAT28-406859 had an MIC value of > 8 $\upmu$g/ml. The MIC_50_ and MIC_90_ values for NAT13-338148 were 1 $\upmu$g/ml and 2 $\upmu$g/ml respectively; both the MIC_50_ and MIC_90_ values for NAT18-355531 and NAT18-355768 were 1 $\upmu$g/ml respectively, similar to vancomycin.

### Antimicrobial activity of the hit scaffolds against gut microflora strains

Contrary to the standard-of-care antibiotics vancomycin and fidaxomicin, which inhibited the Gram-positive Bifidobacterial members at very low concentrations, NAT13-338148 was found to permit their growth even at concentrations > 8 $\upmu$g/ml. NAT18-355531 and NAT18-355768 inhibited the tested *Bacteroides* sp. and *Bifidobacterium* sp. at a slightly lower concentration (4 $\upmu$g/ml) (Table 1B).

**Table 1:** Determination of minimum inhibitory concentration (MIC) values of hit compounds along with controls vancomycin and fidaxomicin.

*C. difficile* strains	NR number	MIC (µg/ml)										
		NAT5-397881	NAT13-331545	NAT13-338148	NAT18-355,531	NAT18-355768	NAT18-356312	NAT27-401005	NAT27-401503	NAT28-406859	Vancomycin	Fidaxomicin
**(A). MIC values against a panel of** ***C. difficile*** **strains**												
I2	NR-13428	> 8	8	1	1	0.5	> 8	> 8	> 8	> 8	1	0.03
I4	NR-13430	> 8	8	0.5	1	1	> 8	> 8	> 8	> 8	0.5	0.015
I6	NR-13432	> 8	8	1	1	0.5	> 8	> 8	> 8	> 8	0.25	0.06
I13	NR-13553	> 8	8	1	1	1	> 8	> 8	> 8	> 8	0.25	0.03
P6	NR-32886	> 8	> 8	0.5	0.5	1	> 8	> 8	> 8	> 8	0.125	0.03
P7	NR-32887	> 8	8	0.5	0.5	0.25	> 8	> 8	> 8	> 8	0.5	0.03
P9	NR-32889	> 8	8	1	1	1	> 8	> 8	> 8	> 8	1	0.03
P19	NR-32895	> 8	> 8	1	0.5	0.5	> 8	> 8	> 8	> 8	1	0.03
P30	NR-32904	> 8	> 8	0.25	1	1	> 8	> 8	> 8	> 8	0.25	0.06
Isolate 20,100,502	NR-49277	> 8	> 8	2	2	2	> 8	> 8	> 8	> 8	0.25	0.06
Isolate 20,100,207	NR-49278	> 8	8	2	1	1	> 8	> 8	> 8	> 8	0.25	0.125
Isolate 20,110,999	NR-49286	> 8	> 8	2	1	1	> 8	> 8	> 8	> 8	0.25	0.25
Isolate 20,110,870	NR-49288	> 8	> 8	2	1	1	> 8	> 8	> 8	> 8	1	0.125
Isolate 20,120,187	NR-49290	> 8	> 8	2	1	1	> 8	> 8	> 8	> 8	1	0.06
ATCC BAA 1870		> 8	> 8	1	1	1	> 8	> 8	> 8	> 8	1	0.06
ATCC 43255		> 8	> 8	0.5	1	1	> 8	> 8	> 8	> 8	1	0.06
MIC_50_		> 8	> 8	1	1	1	> 8	> 8	> 8	> 8	0.5	0.06
MIC_90_		> 8	> 8	2	1	1	> 8	> 8	> 8	> 8	1	0.125

Gut microflora strains	MIC $(\upmu$ g/ml)											
	NAT13-338148	NAT18-355531	NAT18-355768	Vancomycin	Fidaxomicin							
**(B). MIC values against representative members of the human gut microflora**												
*Bacteroides fragilis* HM 20	> 8	4	4	> 8	> 8							
*Bacteroides fragilis* HM 709	> 8	4	4	> 8	> 8							
*Bacteroides fragilis* HM 710	> 8	4	4	> 8	> 8							
*Bacteroides fragilis* HM 711	> 8	4	4	> 8	> 8							
*Bacteroides fragilis* HM 714	> 8	4	4	> 8	> 8							
*Bacteroides dorei* HM 719	> 8	4	4	> 8	> 8							
*Bifidobacterium adolescentis* HM 633	> 8	4	4	0.5	< 0.06							
*Bifidobacterium longum* subsp. *longum* HM 845	> 8	4	4	0.5	< 0.06							
*Bifidobacterium longum* subsp. *longum* HM 846	> 8	4	4	0.5	< 0.06							
*Bifidobacterium longum* subsp. *longum* HM 847	> 8	4	4	0.5	< 0.06							
*Bifidobacterium longum* subsp. *longum* HM 848	> 8	4	4	0.5	< 0.06							
*Bifidobacterium angulatum* HM 1189	> 8	4	4	1	< 0.06							
*Lactobacillus reuteri* HM 102	> 8	> 8	> 8	> 8	> 8							

### Cytotoxic potential of the hit scaffolds

**Figure 2:**
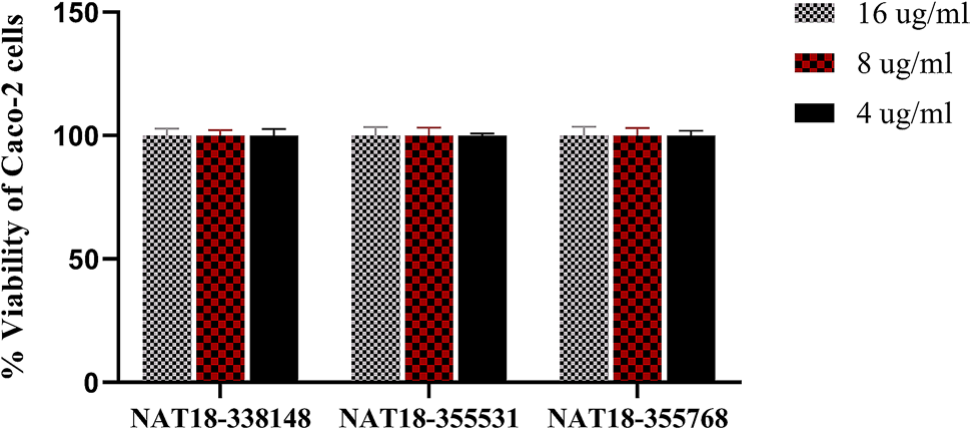
Cytotoxicity assay of hit scaffolds against human colorectal adenocarcinoma (Caco-2) cell line. Percent viable Caco-2 cells measured as ratio of average absorbance relative to DMSO for analyzing cytotoxicity of the hit natural product-derived small molecules at 16 $\upmu$g/ml using the MTS 3-(4,5-dimethylthiazol-2-yl)-5-(3-carboxymethoxyphenyl)-2-(4-sulfophenyl)-2*H*-tetrazolium) assay. DMSO was used as a negative control to determine a baseline measurement for the cytotoxic impact of each compound. The absorbance values represent an average of a minimum of three samples analyzed for each compound. Error bars represent standard deviation values for the absorbance.

To discern the cytotoxic effect of the natural product derived small molecules, the molecules were screened against Caco-2 cells using the MTS assay. Figure 2 represents the results garnered. All the three hit compounds (NAT13-338148, NAT18-355531, and NAT18-355768) were found to be nontoxic to Caco-2 cells at a concentration of 16 $\upmu$g/ml.

## Discussion

*C. difficile* is a common cause of diarrhea, mainly affecting hospitalized patients, and is an increasing health threat worldwide^1,12^. A predisposing factor to CDI is the use of antibiotics for unrelated disease conditions that disrupts the intestinal microbiome causing a state permitting *C. difficile* growth and colonization^13,14^. In spite of antibiotics paving the way for CDI, the standard-of-care therapeutics are limited to antibiotics vancomycin and fidaxomicin, with metronidazole being recommended only in settings where there is limited access to the other two drugs^6^. A major limitation of these first-line antimicrobials is that they fail to assure sustained clinical cure and patients often suffer from recurrent CDI. The transplantation of fecal microbiota has recently been accepted as a potential intervention to tackle this problem of recurrence. However, the use of FMT is accompanied with an increased risk of exposure to organisms of concern^15,16^. The significant drawbacks of the current treatment repertoire necessitate an alternative paradigm for treating CDI with minimal effect on the indigenous intestinal microflora.

HTS is an enabling approach that can be exploited for the discovery of novel scaffolds and can be used as a starting point for drug discovery^17^. Herein, we used this tool to screen a library of 5000 natural product-like compounds against *C. difficile*. In our first screening, we found 34 out of the 5000 compounds with inhibitory activity against the pathogen. To validate this, we did a plate cherry-picking assay which confirmed the anticlostridial activity of 10 of the compounds out of the 34 hits obtained from the initial screening.

Based on the promising results, we sought to decipher the actual concentration of the compounds that can inhibit the pathogen. We did an MIC assay against a panel of 16 hypervirulent and clinically toxigenic *C. difficile* strains. Out of the 10 hits, we found 2 compounds with MIC_90_ values (NAT18-355531 and NAT18-355768) comparable to the MIC_90_ of the first-line drug vancomycin (1 $\upmu$g/ml). The MIC_90_ of the other compound, NAT18-338148, was only onefold greater than that of vancomycin.

A hall mark of CDI remains the recurrence of infection in spite of successful treatment of the initial episode. The absence of the healthy gut microbiome favors the colonization of the intestine by such enteric pathogens. Hence, it is crucial to seek a scaffold that has minimal effects on the human gut microbiome. Contrary to vancomycin and fidaxomicin, NAT13-338148 was found to have no inhibitory effect on the gut microbial species at the tested concentration (8 $\upmu$g/ml). NAT18-355531 and NAT18-355768 were found to not inhibit the *Bacteroides* and *Bifidobacterium* sp. at a concentration of 4 $\upmu$ g/ml.

Evaluating cytotoxicity of novel chemical entities is an approach adopted to increase the probability of success in preclinical animal studies^18^. In this study, we analyzed the cytotoxicity of the natural product- inspired small molecules against Caco-2 cells. The hit scaffolds had no deleterious effects on the Caco-2 cells when treated a concentration of 16 g$\upmu$/ml for 24 h.

Our results demonstrate three novel natural product-like compounds, NAT13-338148, NAT18-355531, and NAT18-355768, with potent in vitro anticlostridial activity. Further studies including synthesis of analogues, investigating their pharmacological parameters and determining the efficacy of the lead compounds and their synthesized analogues in a primary and recurrent CDI mice model will be needed to yield a therapeutic capable of reducing short-term diarrhea and long-term sequelae of recurrent CDI.

## Materials and methods

### Bacterial strains, cell line, and reagents

*C. difficile* isolates were obtained from the American Type Culture Collection (ATCC, Manassas, VA), the Biodefense and Emerging Infections Research Resources Repository (BEI Resources, Manassas, VA), and Microbiologics Inc (St Cloud, Minnesota). The strains were cultured in brain heart infusion broth (BHIS; brain heart infusion medium from Becton, Dickinson and Company, Cockeysville, MD), supplemented with yeast extract (Fisher Scientific, Waltham, MA), L-cysteine (Alfa Aesar, Haverhill, MA), resazurin, vitamin K1, and hemin (Sigma-Aldrich, St. Louis, MO)^19,20^. Caco-2 cell line was purchased from ATCC. Dulbecco’s modified Eagle’s medium (DMEM) and penicillin/ streptomycin were obtained from Sigma-Aldrich (St. Louis, MO)., fetal bovine serum (FBS), Phosphate- buffered saline (PBS), and non-essential amino acids (NEAA) were purchased from Fisher Scientific (Waltham, MA). MTS 3-(4,5-dimethylthiazol-2-yl)-5-(3-carboxymethoxyphenyl)-2-(4-sulfophenyl)-2*H*-tetrazolium) reagent was procured from Promega (Madison, WI).

### Libraries and control antibiotics

The AnalytiCon NATx library containing 5000 natural product-like synthetic compounds was purchased from AnalytiCon Discovery (Potsdam, Germany) by the Chemical Genomics facility at the Purdue Institute of Drug Discovery. The compounds were provided in sixteen 384-well plates as 1 mM DMSO stock. Hit compounds were further purchased from AnalytiCon Discovery. Vancomycin hydrochloride (Gold Biotechnology, Olivette, MO), metronidazole (Alfa Aesar), and fidaxomicin (Cayman Chemicals) were purchased from commercial vendors.

### High-throughput screen (HTS) and plate cherry-picking

*C. difficile* ATCC BAA 1870 was grown on BHIS agar plate supplemented with yeast extract, resazurin, hemin, vitamin K, and L-cysteine and incubated anaerobically at 37 °C for 48 h. Prior to screening, compounds (180 nL of 1 mM stock solutions) were arrayed into clear 384-well plates with negative control (DMSO) and positive controls (vancomycin, metronidazole, and fidaxomicin) using an Echo acoustic dispenser. *C. difficile* ATCC BAA 1870 colonies were suspended in sterile PBS and adjusted to the turbidity of 0.5 McFarland solution. An approximate 150 $\upmu$L of the PBS containing bacteria was transferred to 40 ml of freshly prepared BHIS broth to attain a bacterial concentration of approximately 5 × 10^5^ CFU/ml. 60 $\upmu$L of this solution was transferred to each well of the 384-well assay plate using an automatic dispenser, thus bringing the final concentration of the compounds to 3 M$\upmu$. The plates were then incubated anaerobically for 48 h at 37 °C. After incubation, the OD_600_ was determined using SpectraMax i3 Multi-Mode Microplate Reader (Molecular Devices, Sunnyvale, CA). The Z’ value was calculated using the equation Z’ = 1 − [(3 $\sigma$_p_ + 3 $\sigma$_n_)/(µ_p_ − µ_n_)], where $\sigma$ is the SD, µ is the average, p indicates the antibiotic-treated control, and n indicates the DMSO control and plates with Z’ < 0.5 were repeated^21^. Hits were further verified by calculating the percent of cell growth inhibition and compounds exhibiting $\ge 95\%$ were selected for plate cherry-picking. Percent of cell growth inhibition was plotted using GraphPad Prism v 8.0.

### Minimum inhibitory concentration (MIC) assay against a panel of *C. difficile* strains

The hit compounds identified from plate cherry-picking were ordered from AnalytiCon Discoveries. The compounds were dissolved in DMSO, and the minimum inhibitory concentration (MIC) was determined as described previously^22–26^. Briefly, 16 *C. difficile* clinical isolates were used to prepare a bacterial suspension equivalent to the turbidity of 0.5 McFarland solution, and added to BHIS broth to attain a bacterial concentration of 5 × 10^5^ CFU/ml. Hit compounds validated via cherry picking were added at a concentration of 8 g$\upmu$/ml to the first row of the 96-well plate. Serial dilution was carried out and the plates were incubated anaerobically at 37ºC for 48 h. The MIC was defined as the lowest concentration of the drugs that inhibited bacterial growth after the incubation period of 48 h.

### MIC assay against gut microflora

The activity of the hit compounds (Table S1) was further verified against the 13-gut microflora (Table S3) as described in section “Minimum inhibitory concentration (MIC) assay against a panel of *C. difficile* strains”. Briefly, the hit compounds (concentration = 8 g$\upmu$/ml) were added to the first row of the 96-well plate. Bacterial suspension of *Bacteroides* sp. and *Bifidobacterium* sp. in BHIS broth and *Lactobacillus* sp. in MRS broth were prepared to attain a bacterial concentration of 5 × 10^5^ CFU/ml. The diluted bacterial suspension (in BHIS and MRS broth respectively) was used for serial dilution and plates were incubated at 37ºC for 48 h. MIC values were recorded following the incubation period.

### Cytotoxicity assay

To evaluate the potential toxic effect of the natural product-like synthetic compounds, cytotoxicity assay was performed against Caco-2 cells as has been described previously^27–30^. Caco-2 cells were cultured in DMEM supplemented with 10% FBS, 1% penicillin/streptomycin, and 1% NEAA and incubated at 37 °C in presence of 5% CO_2_. Drugs were added at a starting concentration of 16 g$\upmu$/ml and the control wells received DMSO alone at a concentration equal to that in drug-treated cell samples. The cells were incubated with the compounds in a 96-well plate for 24 h prior to addition of the assay reagent MTS. Absorbance readings at OD_490_ were taken using a SpectraMax i3 Multi-Mode Microplate Reader (Molecular Devices, Sunnyvale, CA). Cell survival post treatment was plotted as percentage viability of drug-treated cells when compared to the DMSO-treated control cells using GraphPad Prism v 8.0.

## Supplementary Information

Supplementary Information.
