# Supplementary material for: High-throughput screening identifies a novel natural product-inspired scaffold capable of inhibiting Clostridioides difficile in vitro
Source: Sci Rep. 2021 May 25;11:10913. doi: 10.1038/s41598-021-90314-3 (PMC8149678; doi:10.1038/s41598-021-90314-3)
Supplement: Supplementary file 1 — Supplementary Information. [file 41598_2021_90314_MOESM1_ESM.docx]

**Supplementary information**

**TableS1: Structure and IUPAC names of the hit compounds.**

| **ID number** | **Structure** | **Formulae** | **Mol. wt** |
| --- | --- | --- | --- |
| **NAT13-338148** |  | C_28_H_31_N_5_ | 437.579 |
| **NAT18-355531** |  | C_21_H_19_ClF_3_N_5_O_2_ | 465.856 |
| **NAT18-355768** |  | C_20_H_19_F_3_N_6_O_2_ | 432.399 |
| **NAT28-406859** |  | C_27_H_42_N_4_O_2_ | 454.648 |
| **NAT18-356312** |  | C_21_H_22_FN_5_O_2_ | 395.43 |
| **NAT27-401005** |  | C_24_H_32_N_4_O_5_ | 456.535 |
| **NAT5-397881** |  | C_23_H_36_N_4_O_3_ | 416.557 |
| **NAT27-401503** |  | C_24_H_32_N_4_O_5_ | 456.535 |
| **NAT13-337317** |  | C_18_H_23_F_3_N_2_O_3_ | 372.382 |
| **NAT13-331545** |  | C_27_H_29_F_3_N_4_O_3_ | 514.539 |

**Table S2: List of *C. difficile* strains used in the study.**

| **Bacterial strains / ID number** | **Source and comments** |
| --- | --- |
| Isolate 2/ NR-13428 | Isolated in 2008/2009 from a patient diagnosed with CDI in the Mid-Atlantic region of the USA |
| Isolate 4/ NR-13430 | Isolated in 2008/2009 from a patient diagnosed with CDI in the Mid-Atlantic region of the USA |
| Isolate 6/ NR-13432 | Isolated 2008/2009 from a patient diagnosed with CDI in the Mid-Atlantic region of the USA |
| Isolate 13/ NR-13553 | Isolated 2008/2009 from a patient diagnosed with CDI in the Mid-Atlantic region of the USA |
| P6/ NR-32886 | Toxigenic strain procured in 2001from fecal matter of a patient suffering from recurrent *C. difficile* infection in western Pennsylvania, USA |
| P7/ NR-32887 | Toxigenic strain procured in 2001from fecal matter of a patient with *C. difficile* infection in western Pennsylvania, USA |
| P9/ NR-32889 | Toxigenic strain procured in 2001from fecal matter of a patient suffering from recurrent *C. difficile* infection |
| P19/ NR-32895 | Toxigenic strain procured from fecal matter of a patient suffering from recurrent *C. difficile* infection in western Pennsylvania, USA in 2005 |
| P30/ NR-32904 | Isolated in 2009 from fecal matter of an asymptomatic human patient in western Pennsylvania, USA |
| Isolate 20100502/ NR-49277 | Isolated from the fecal matter of an elderly male patient diagnosed with community-associated (CA) *C. difficile* infection in Colorado, USA in the year of 2010 |
| Isolate 20100207/ NR-49278 | Isolated from the stool of an elderly adult male patient diagnosed with healthcare-associated (HA) *C. difficile* infection in New York, USA in the year of 2010 |
| Isolate 20110999/NR-49286 | Isolated from the stool sample of an elderly female patient diagnosed with healthcare-associated (HA) *C. difficile* infection in western/midwestern, USA in the year of 2011 |
| Isolate 20110870/ NR-49288 | Isolated from the stool sample of a young adult female patient diagnosed with healthcare associated (HA) *C. difficile* infection in Tennessee, USA in the year of 2011 |
| Isolate 20120187/ NR-49290 | Isolated from the stool sample of an elderly adult male patient with healthcare-associated (HA) *C. difficile* infection in Tennessee, USA in the year of 2011 |
| ATCC BAA 1870 | Classified as toxinotype IIIB, ribotype 027, presence of *cdtB*^a^, *tcdA*^b^, and *tcdB*^c^ genes |
| ATCC 43255/ VPI 10463 | Ribotype 087, presence of *tcdA*^b^ and *tcdB*^c^ genes |

^a^ cdtB= *C. difficile* binary toxin

^b^ tcdA= *C. difficile* toxin A gene

^c^ tcdB= *C. difficile* toxin B gene

**Table S3: List of gut microflora strains used in this study.**

| **Bacterial strains** | **Source and comments** |
| --- | --- |
| *Bacteroides fragilis* HM 20 | Isolated from the transverse colon of a healthy female in Alberta, Canada |
| *Bacteroides fragilis* HM 709 | Isolated in Massachusetts, USA from the feces of a healthy adult |
| *Bacteroides fragilis* HM 710 | Isolated in Massachusetts, USA from the feces of a healthy adult |
| *Bacteroides fragilis* HM 711 | Isolated in Massachusetts, USA from the feces of a healthy adult |
| *Bacteroides fragilis* HM 714 | Isolated in Massachusetts, USA from the feces of a healthy adult |
| *Bacteroides dorei* HM 719 | Isolated in Boston, Massachusetts, USA from the feces of a healthy adult |
| *Bifidobacterium adolescentis* HM633 | Isolated in 1996 from the fecal sample of a healthy two-year old in Aberdeen, Scotland, UK. |
| *Bifidobacterium longum* subsp. *longum* HM 845 | Isolated from a one-year old human patient |
| *Bifidobacterium longum* subsp. *longum* HM 846 | Isolated from the feces of a six-year old child in Russia in 2006 |
| *Bifidobacterium longum* subsp. *longum* HM 847 | Isolated from a one-year old patient |
| *Bifidobacterium longum* subsp. *longum* HM 848 | Isolated from a six-year old patient |
| *Bifidobacterium angulatum* HM 1189 | Isolated from human stool in Guelph, Ontario, Canada |
| *Lactobacillus reuteri* HM 102 | Isolated from the feces of a healthy Finnish child |
